# Development of Ag_0.04_ZrO_2_/rGO heterojunction, as an efficient visible light photocatalyst for degradation of methyl orange

**DOI:** 10.1038/s41598-022-16673-7

**Published:** 2022-07-19

**Authors:** Rana Muhammad Arslan Iqbal, Tehmina Akhtar, Effat Sitara, Habib Nasir, Aliya Fazal, Uzaira Rafique, Sharif Ullah, Adeel Mehmood

**Affiliations:** 1 Department of Chemistry, School of Natural Sciences, National University of Sciences and Technology, H-12, Islamabad, 44000 Pakistan; 2 Department of Chemistry, Fatima Jinnah Women University, Rawalpindi, Pakistan; 3 Department of Environmental Sciences, Fatima Jinnah Women University, Rawalpindi, Pakistan; 4 Department of Chemical Engineering and Energy Systems Research, Ajou University, Suwon-si, Gyeonggi-do 16499 Republic of Korea

**Keywords:** Chemistry, Nanoscience and technology

## Abstract

Methyl orange (MO) is mutagenic, poisonous, and carcinogenic in nature, hence, effective methods are required for its degradation. We have synthesized pure ZrO_2_, Ag-doped ZrO_2,_ and Ag-doped ZrO_2_/rGO as hybrid photocatalysts by facile hydrothermal method. These photocatalysts were characterized by powder XRD, scanning electron microscopy, EDX, FTIR, photoluminescence, UV–Vis diffuse reflectance (DRS), and Raman spectroscopy. The photodegradation of MO (10 ppm) was studied with pure ZrO_2_, Ag-doped ZrO_2,_ and Ag-doped ZrO_2_/rGO (10 mg/100 mL catalyst dosage) photocatalysts at 100 min irradiation time under UV–Visible light. The pH effect and catalyst dosage on photodegradation of MO was investigated. Ag_0.04_ZrO_2_/rGO photocatalyst exhibited the maximum photocatalytic degradation of MO (87%) as compared to Ag_0.04_ZrO_2_ (60%) and pure ZrO_2_ (26%). Reusability experiments ensured the excellent stability of photocatalyst after five consecutive experiments. To the best of our knowledge, this is the first report on the facile hydrothermal synthesis of Ag_0.04_ZrO_2_/rGO photocatalyst for photocatalytic degradation of methyl orange.

## Introduction

Environmental contamination, particularly water contamination, has become one of the most pressing challenges in recent years. Diverse toxins from industrial effluents build up in the water, posing a threat to humans, animals, microbes, and aquatic life^1^. The dyes, which are organic in origin, are among the pollutants that give water color. Furthermore, according to the World Bank, the textile industry contributes 17–20 percent of industrial water pollution through dyes^2^. The majority of dyes, including methyl orange are comprised of azoic dyes, which have a nitrogen π-bond in their structure. The azo dye methyl orange (MO) is one of the most widely used dyes in the textile, food, leather, and pharmaceutical industries. For the detection of hydrogen gas and hydrochlorides, MO is also utilized as a coloring agent^3^. The addition of MO to water is a major source of worry since it has a significant impact on water quality and creates dangerous scenarios for aquatic life. The toxicity, mutagenesis, and carcinogenic characteristics of MO are the most concerning features of its use^4^. Because MO is difficult to degrade; selective approaches are required^5^. Coagulation, reverse osmosis, membrane filtering, oxidation, reduction, complexometric, ion exchange, anaerobic, and aerobic techniques are all commonly employed for MO degradation. Among these techniques, photocatalytic degradation appears to be the most promising for MO degradation^6,7^. Hence, many oxides and sulfides of semiconductors such as TiO_2_, SnO_2_, Fe_2_O_3_, ZnO, WO_3_, CdS, WS_2_, ZnS, and MoS_2_, as well as their binary and ternary^8,9^ mixed oxides or sulfides have been reported for the photodegradation of organic pollutant like antibiotics^10–13^ and dyes^14–22^.

ZrO_2_, a cost-effective and nontoxic transition metal oxide, has high thermal and chemical stability, low thermal conductivity, and high corrosion resistance^23^. Controllable morphology, mesoporous structure, and crystallinity make nanosized ZrO_2_ an active photocatalyst as it enhances the light absorption capability enabling reactants to approach surface active sites mesoporous structure. So far, ZrO_2_ has been widely studied due to its relatively wide bandgap values (3.25–5.1 eV) and highly negative conduction band potential. However, intrinsic ZrO_2_ with such a wide band gap is found to be only responsive to ultraviolet (UV) light, which is impracticable for the use of visible light. For the best use of solar energy ZrO_2_ lattice incorporated with a suitable metal on a conducting substrate is widely used to form an impurity state, which can shift its absorption edge into the visible light region. Reduced graphene oxide (rGO) a conducting substrate, acts as an excellent electron mediator, integrating rGO with photocatalysts increases the surface area hence the absorption capacity of the catalyst which resultantly improves electron transport^24^. The combination of a reduced conducting substrate and photocatalyst reduces the bandgap energy, enhances visible light absorption, stabilizes nanocomposite, and enables electron–hole separation throughout the heterojunction^25–27^. To the best of our knowledge, the synthesis of Ag-doped ZrO_2_/rGO is not reported yet.

This study aims to synthesize the silver doped ZrO_2_/rGO photocatalysts and degrade methyl orange (MO) under visible light. In the current study, we have synthesized the photocatalysts by facile hydrothermal method. This is the first report on the facile hydrothermal synthesis of Ag_0.04_ZrO_2_/rGO photocatalyst to the best of our knowledge. These photocatalysts were characterized by powder XRD, SEM, EDX, FTIR, photoluminescence (PL), EPR, and UV–Vis diffuse reflectance (DRS). The photodegradation of MO with ZrO_2_, Ag-doped ZrO_2,_ and Ag-doped ZrO_2_/rGO was evaluated under visible light. Ag_0.4_ZrO_2_/rGO photocatalyst exhibits the highest catalytic activity among the prepared catalysts.

## Results and discussion

### X-ray diffraction (XRD)

**Figure 1:**
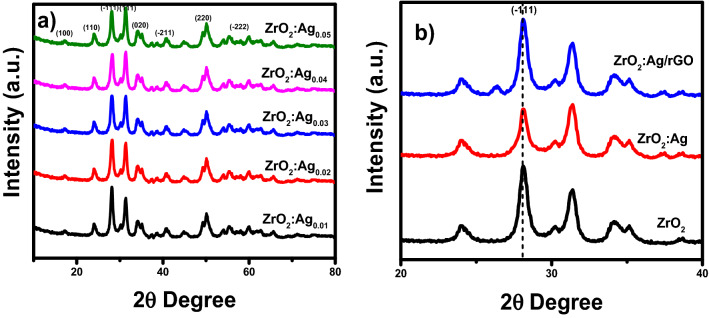
(**a**) XRD patterns of Ag_x_ZrO_2_ (x = 0.01–0.05), (**b**) comparison of XRD patterns of Ag_0.04_ZrO_2_/rGO and Ag_0.04_ZrO_2_ with pure ZrO_2_.

The XRD spectra of the Ag_-_doped ZrO_2_ (x = 0.01–0.05) are shown in Fig. 1a. The XRD patterns of Ag_x_ZrO_2_ (x = 0.01–0.05) photocatalysts are similar to that of ZrO_2_ and no peak is observed for Ag. The XRD pattern of Ag_x_ZrO_2_ (x = 0.01–0.05) shows the well-defined diffraction peaks corresponds to the monoclinic phase of ZrO_2_ at 17.5° (100), 24.3° (110), 28.3° (− 111), 31.5° (111), 34.3° (020), 40.7°, (− 211), 50.3° (220), 58.2° (− 222), 63° (311), 65.9° (− 231) attributing to crystal planes with the JCPDS file No. 65-1023. The only difference between the XRD patterns is the decrease in intensity of a characteristic peak of zirconia at 28.3°, with the increase in the amount of Ag. Figure 1b shows the comparison of XRD pattern of Ag_0.04_ZrO_2_/rGO and Ag_0.04_ZrO_2_ with pure ZrO_2_, indicating that Ag is intrinsically doped in ZrO_2_ which improves the catalytic activity of the photocatalyst^28^.

### SEM and EDX study

**Figure 2:**
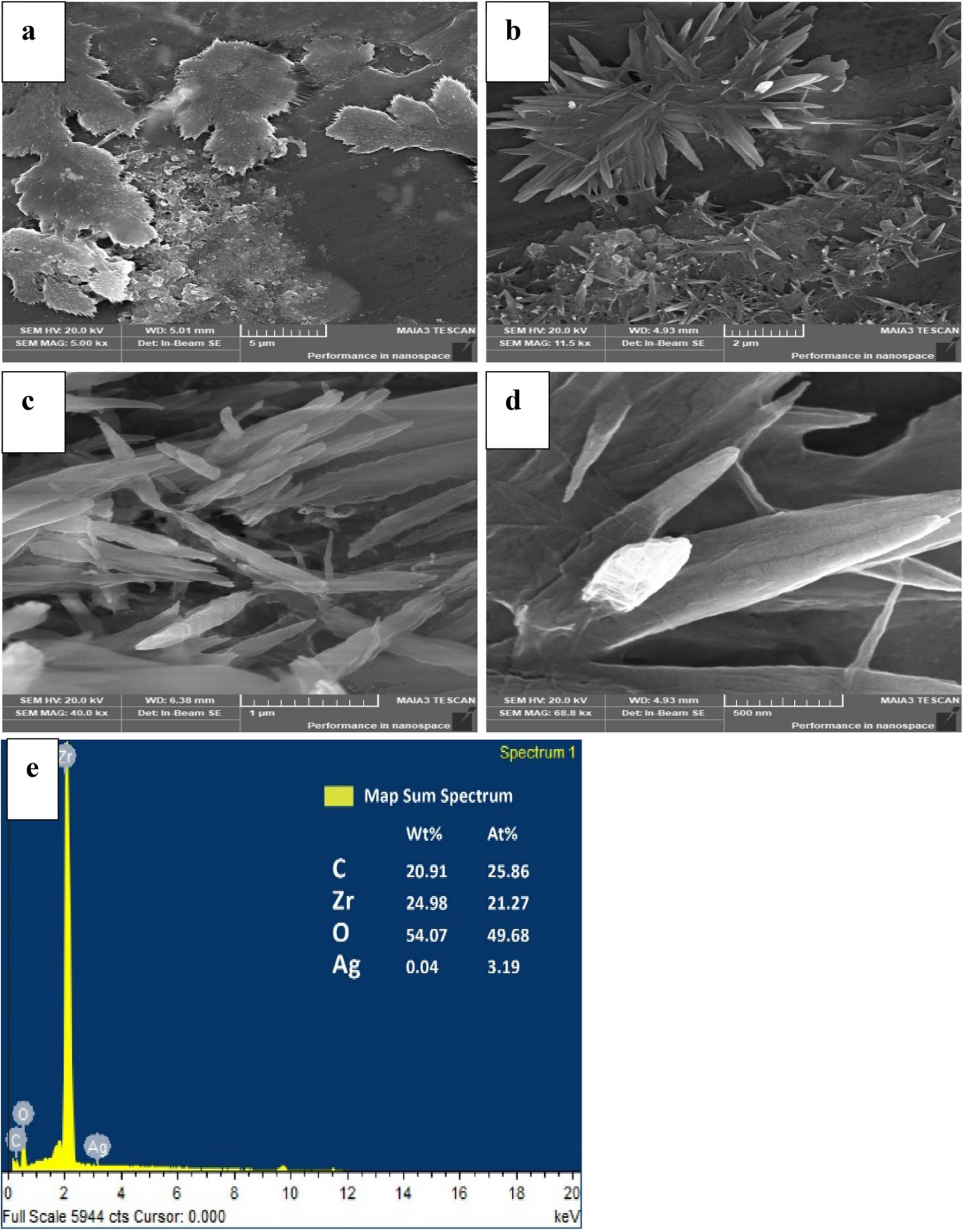
(**a**–**d**) Scanning electron micrographs of Ag_0.04_ZrO_2_/rGO and (e) EDX of Ag_0.04_ZrO_2_/rGO.

The SEM micrographs of pure ZrO_2_ are shown in Fig. S1a–c and EDX are shown in Fig. S1d. Pure ZrO_2_ shows the large-sized cavities of thick rod-like structure^29,30^. The SEM micrographs of Ag_0.04_ZrO_2_ are shown in Fig. S2a–d which shows that the crystallinity has decreased with increasing concentration of Ag and EDX is in Fig. S2e. The crystallinity is regained when the heterostructure with rGO is formed. The SEM micrographs of Ag_0.04_ZrO_2_/rGO photocatalyst are shown in Fig. 2a–d having dense nanorods which are aligned vertically. The average diameter of the nanorods is 100 nm. The large network structure of Ag_0.04_ZrO_2_/rGO nanorods may not only increase the active sites for a photocatalytic reaction but also provide channels for solution diffusion during the intercalation/de-intercalation process toward photocatalyst.

Elemental analysis of Ag_0.04_ZrO_2_/rGO nanorods was done by EDX which is shown in Fig. 2e. The spectrum shows the O, Ag, Zr, and C with an atomic percentage of 49.68, 3.19, 21.27, and 25.86, respectively^31^.

### Fourier transform infrared spectroscopy

FTIR spectroscopy was used to identify chemical bonds as well as functional groups of synthesized material by producing an infrared absorption spectrum. ZrO_2_, Ag_0.04_ZrO_2,_ and Ag_0.04_ZrO_2_/rGO photocatalysts were characterized with FTIR spectroscopy. In Fig. S3a, a comparison of the FTIR spectra of pure ZrO_2_ and Ag_0.04_ZrO_2_ is presented. Both the FTIR spectra show the band around 559 cm^−1^ which arises due to the Zr-O vibration in zirconia. Figure S3b shows the FTIR spectrum of Ag_0.04_ZrO_2_/rGO (1:1). In this spectrum, the band at 561 cm^−1^ is due to the Zr-O vibrations in the photocatalyst. This spectrum also shows the bands of rGO^32^.

### UV–Vis diffuse reflectance spectroscopy

**Figure 3:**
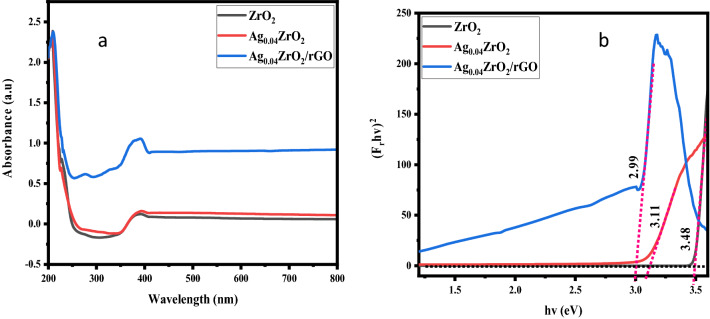
(**a**) UV–Vis DRS spectra of ZrO_2_, Ag_0.04_ZrO_2_, and Ag_0.04_ZrO_2_/rGO photocatalysts. (**b**) Eg of ZrO_2_, Ag_0.04_ZrO_2_, and Ag_0.04_ZrO_2_/rGO photocatalysts.

UV–Vis DRS absorbance of ZrO_2,_ Ag_0.04_/ZrO_2,_ and Ag_0.04_ZrO_2_ /rGO results are displayed in Fig. 3. It can be seen that ZrO_2_ exhibits absorption in the UV region due to its large bandgap. The absorption is red-shifted by doping of Ag into ZrO_2_ as in the case of Ag_0.04_/ZrO_2_ while Ag_0.04_ZrO_2_/rGO shows strong adsorption in the visible region due to the formation of heterostructure which decreases the recombination rate of e^−^/h^+^ pairs. This improved change in absorption of Ag_0.04_ZrO_2_/rGO increases the photocatalytic activity^33^.

The Tauc equation was used to calculate the bandgap energy of the synthesized photocatalysts^34^:

1 $$ \left( {{\text{h}}\nu \alpha } \right)1/{\text{n}} = {\text{A }}\left( {{\text{h}}\nu - {\text{Eg}}} \right) $$

where, h is Planck’s constant, ν is the vibrational frequency, α is the absorption coefficient, Eg is the bandgap energy (eV), A is a proportionality constant, and n refers to the type of electron transition (for directly allowed transitions, n = 1/2). The value of α is directly proportional to the Kubelka– Munk function (F(R∞))^35^:

2 $$ \left( {{\text{h}}\nu {\text{F}}\left( {{\text{R}}\infty } \right)} \right)^{2} = {\text{A}}\left( {{\text{h}}\nu - {\text{Eg}}} \right) $$

The Tauc plot shows the bandgap energy by the projection of the tangent on the x-axis to the turning point of curvature. The result is shown in Fig. 3b.

The bandgap energies of ZrO_2_, Ag_0.04_ZrO_2_, and Ag_0.04_ZrO_2_/rGO are 3.48, 3.11, and 2.99 eV, respectively. The incorporation of Ag as dopant has lowered the bandgap energy of Ag_0.04_ZrO_2_ (3.06 eV) while the addition of rGO has further lowered the bandgap of Ag_0.04_ZrO_2_/rGO thus increasing the photocatalytic activity^36–38^.

### Photoluminescence analysis

**Figure 4:**
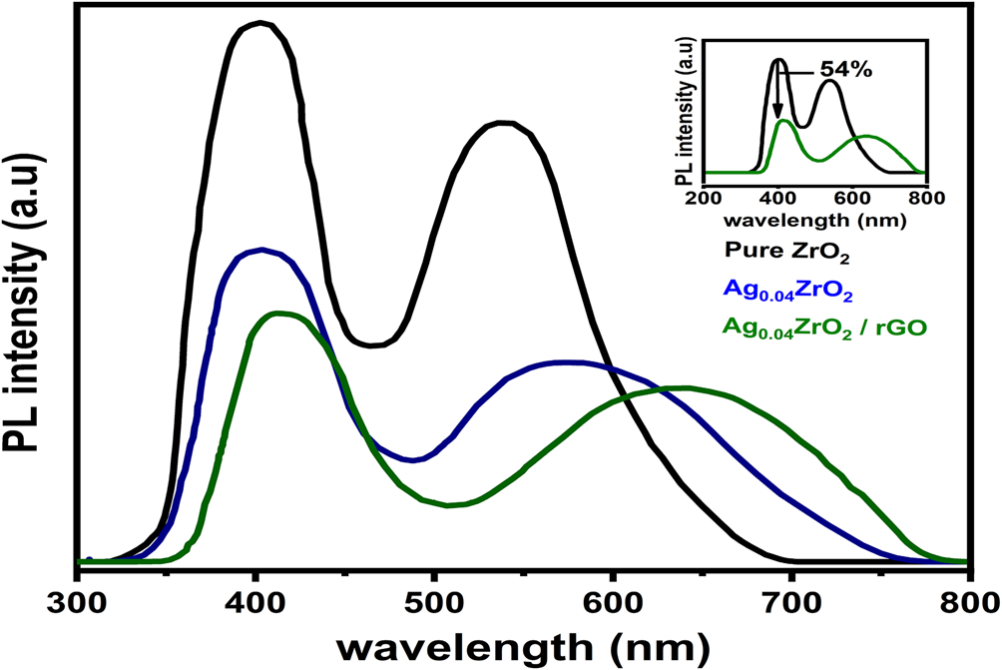
Photoluminescence (PL) spectra of ZrO_2_, Ag_0.04_ZrO_2_, and Ag_0.04_ZrO_2_/rGO.

Photoluminescence (PL) spectroscopy is used to observe the separation and transfer of photogenerated electrons and holes in the photocatalyst/heterojunctions. Figure 4 shows the PL spectra of ZrO_2_, Ag_0.04_ZrO_2_, and Ag_0.04_ZrO_2_/rGO photocatalysts with an excitation wavelength of 325 nm. The shorter and longer wavelength emission of ZrO_2_ and Ag_0.04_ZrO_2_/rGO photocatalysts could result from near-band-edge transitions and oxygen vacancies respectively^39^.

The redshift in the spectrum of Ag_0.04_ZrO_2_/rGO could be attributed due to interfacial charge transfer from Ag_0.04_ZrO_2_ to rGO. This charge transfer decreases the PL intensity of Ag_0.04_ZrO_2_/rGO photocatalyst^40,41^. The intensity is observed in the following order: ZrO_2_ > Ag_0.04_ZrO_2_ > Ag_0.04_ZrO_2_/rGO. The electron/hole pairs are well separated in Ag_0.04_ZrO_2_/rGO, which exhibits higher photocatalytic activity.

### Specific surface area analysis (BET)

**Figure 5:**
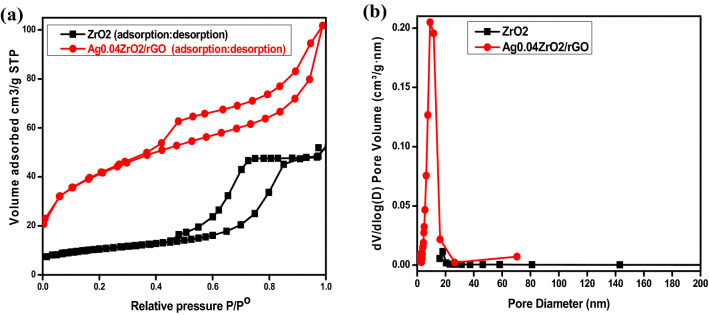
**(a**) Adsorption desorption isotherm for ZrO_2_ and Ag_0.04_ZrO_2_/rGO, (**b**) pore volume and pore diameter of the as prepared pure ZrO_2_ and the composite Ag_0.04_ZrO_2_/rGO.

Figure 5a shows the nitrogen adsorption–desorption studies of ZrO_2,_ and Ag_0.04_ZrO_2_/rGO photocatalysts. These studies are conducted to measure the specific BET surface area and pore structure of the photocatalysts. The BET surface area of Ag_0.04_ZrO_2_/rGO photocatalyst was calculated as 142.441 m^2^/g which is higher than ZrO_2_ which is 37.3996 m^2^/g. An increase in the pore diameter presented in Fig. 5b from 0.08026 cm^3^/g for ZrO_2_ to 0.98852 cm^3^/g for Ag_0.04_ZrO_2_/rGO is also observed. This suggests that the higher surface area and pore volume of Ag_0.04_ZrO_2_/rGO can be achieved by the modification of ZrO_2_ with Ag and rGO. The higher specific BET surface area partly justifies the better adsorption and faster removal of pollutants interacting with the surface of the photocatalyst. Because of higher specific BET surface area, Ag_0.04_ZrO_2_/rGO shows best photocatalytic activity. The specific surface area, mean pore diameter, pore volume, and BHJ pore diameter are summarized in Table 1.

**Table 1:** Summary of the specific surface area, pore volume, mean pore diameter and BHJ pore diameter is presented the table.

	S_BET_ (m^2^/g)	Pore volume (cm^3^/g)	Mean pore diameter (nm)	BHJ pore diameter (nm)
ZrO_2_	37.399	0.08026	8.58445	8.983
Ag0.04ZrO_2_/rGO	142.441	0.98852	9.89842	82.694

### Degradation study of methyl orange (MO)

**Figure 6:**
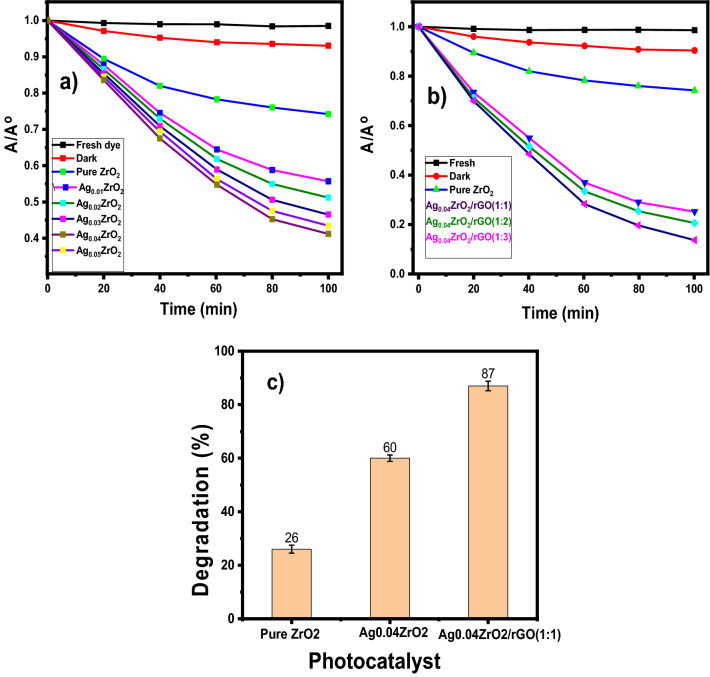
Comparison of degradation of MO (**a**) pure ZrO_2_ and Ag_x_ZrO_2_ (x = 0.01–0.05), (**b**) pure ZrO_2_ and Ag_0.04_ZrO_2_/rGO (1:1 to 1:3) and (**c**) % degradation of MO with pure ZrO_2_, Ag_0.04_ZrO_2_ and Ag_0.04_ZrO_2_/rGO.

The degradation of MO was evaluated under visible irradiation with ZrO_2_, Ag_0.04_ZrO_2_, and Ag_0.04_ZrO_2_/rGO (1:1) photocatalysts are shown in Fig. S4. Figure 6a,b shows the A/A° of MO using ZrO_2_, Ag_x_ ZrO_2_ (x = 0.01–0.05), and Ag_0.04_ZrO_2_/rGO (1:1, 1:2 and 1:3) photocatalysts under visible radiations. Figure S5 shows the comparison of A/A° of degradation of MO ZrO_2_, Ag_0.04_ZrO_2,_ and Ag_0.04_ZrO_2_/rGO. Figure 6c shows the % degradation of MO with pure ZrO_2_, Ag_0.04_ZrO_2,_ and Ag_0.04_ZrO_2_/rGO. The Ag_0.04_ZrO_2_/rGO exhibits 87% degradation while Ag_0.04_ZrO_2_ and ZrO_2_ show 60% and 26% degradation of MO in 100 min_._ The degradation of MO is highest with Ag_0.04_ZrO_2_/rGO photocatalyst as compared to Ag_0.04_ZrO_2_ and ZrO_2_ due to lower bandgap energy and a lower rate of recombination of e^−^/h^+^ in Ag_0.04_ZrO_2_/rGO. Figure S6 shows the comparison of % degradation of MO (a)ZrO_2_ and Ag_x_ZrO_2_ x = 0.01 to 0.05 (b) ZrO_2_ and Ag_0.04_ZrO_2_/rGO (1:1, 1:2 and 1:3 ) photocatalysts.

### Kinetic studies

**Figure 7:**
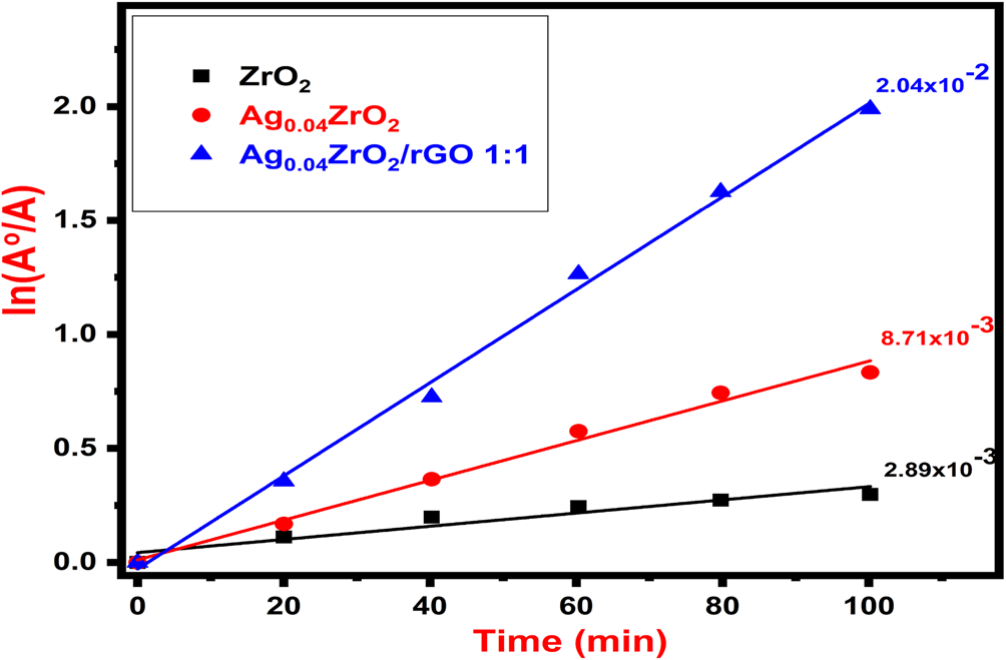
Reaction kinetics of degradation of MO with ZrO_2_, Ag_0.04_ZrO_2,_ and Ag_0.04_ZrO_2_/rGO.

The photocatalytic degradation follows a pseudo-first-order kinetic reaction; its kinetics can be expressed as follows:

$$ \ln ({{A_{o} } \mathord{\left/ {\vphantom {{A_{o} } {A_{t} }}} \right. \kern-\nulldelimiterspace} {A_{t} }}) = k $$

where *k* is the reaction rate constant and *t* is the reaction time. Figure 7 shows the reaction kinetics of degradation of MO by ZrO_2_, Ag_0.04_ZrO_2,_ and Ag_0.04_ZrO_2_/rGO (1:1) photocatalysts.

These results illustrate that MO is degraded by Ag_0.04_ZrO_2_/rGO more efficiently than pure ZrO_2_ or Ag_0.04_ZrO_2_. The degradation rate constant (*k*) is calculated from the slope of the straight line. The degradation rate constant of Ag_0.04_ZrO_2_/rGO with 1:1 (0.0204) is higher than that of doped Ag_0.04_ZrO_2_ (0.00871) and pure ZrO_2_ (0.00289).

### Effect of pH on the photocatalytic performance

**Figure 8:**
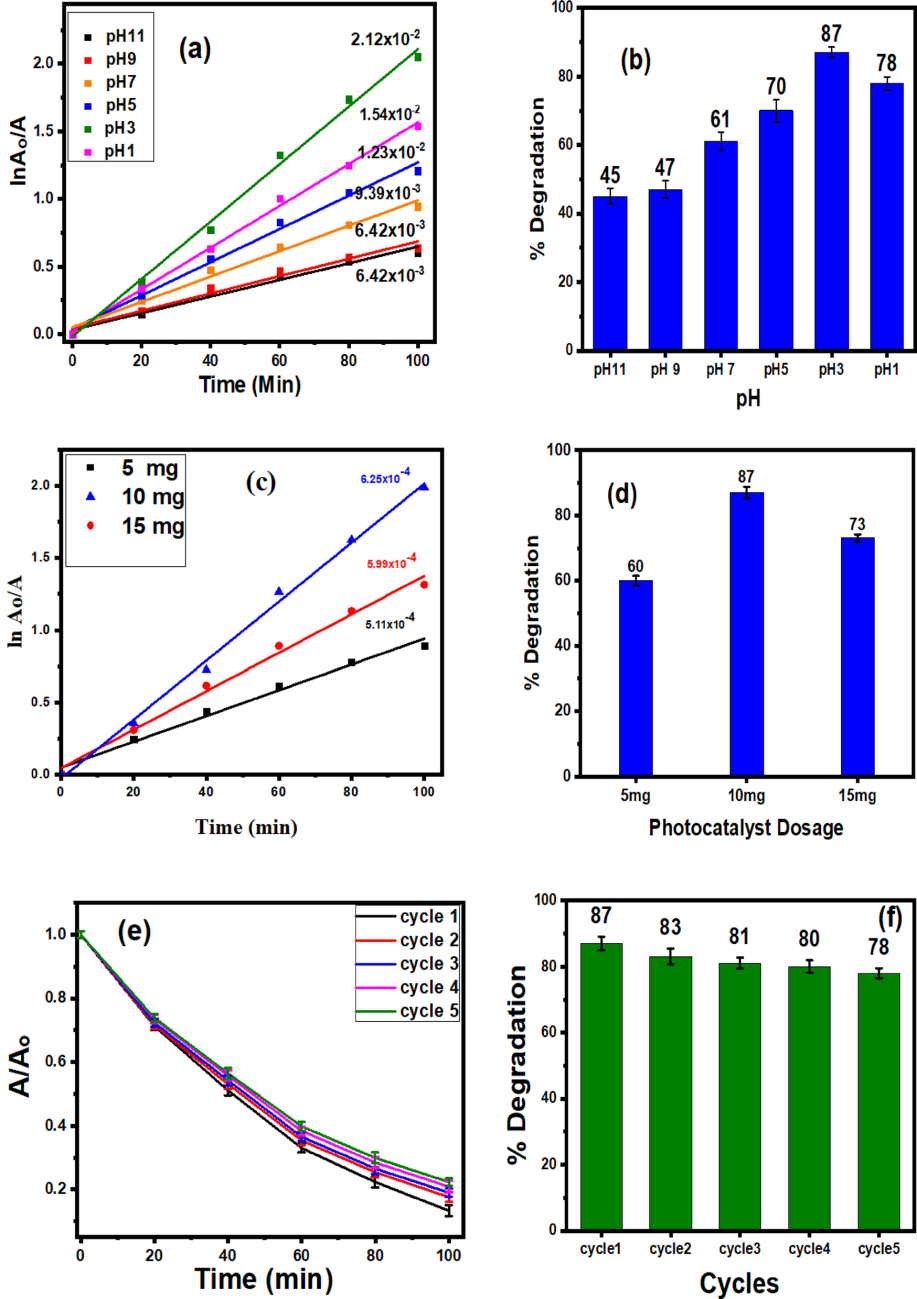
Photocatalytic degradation of MO (**a**) kinetic simulation, (**b**) bar graph % degradation at various pH, (**c**) Kinetic simulation, (**d**) Bar graph of % degradation at different catalyst dosage, (**e**) Reusability of Ag_0.04_ZrO_2_/rGO up to 5 cycles, (**f**) Bar graph of recycling.

The pH is a major factor that affects the surface charge of the photocatalyst, the nature of the dye, and the ability of the dye to absorb into the photocatalyst surface. The degradation of MO was performed at pH 1,3,5,7,9 and 11 at a fixed dose of Ag_0.04_ZrO_2_/rGO (Fig. 8a,b). The degradation of MO is higher in acidic pH and is less in basic pH. However, under acidic conditions, MO change to a quinone structure. A visible color change, along with an absorbance peak shift was observed at lower pH values, further supporting the existence of a quinone structure of MO. The quinone structure is more prone to oxidation over the azo structure due to the sulfonic groups (–SO_3_^−^) aiding in capturing hydrogen and further enhancing the hydrophobicity of the catalyst surface^42^. The enhanced degradation of MO at lower pH 03 is due to the formation of hydroxyl radicals during the reaction (OH^−^ + h^+^ → OH^**·**^), the hydroxyl radicals are scavenged more slowly at a lower pH allowing them to react more readily with the dye.

### Effects of dosage of the catalyst

To examine the effect of dosage of photocatalyst, different experiments were performed at 10 ppm MO concentration and pH 3, by varying the dose of Ag_0.04_ZrO_2_/rGO photocatalyst between 5 and 15 mg/100 mL. It can be seen in Fig. 8c,d that the degradation rate of MO increases with the increasing dosage of Ag_0.04_ZrO_2_/rGO. However, it is interesting to find that the degradation rate first increased with the increased dosage of catalyst (5–10 mg), then decreased with the further increase of catalyst (15 mg). The reason is that by increasing the catalyst dosage the surface area of the catalyst for the adsorption of MO increases which increases the MO degradation. But when the catalyst dosage is increased to 15 mg, a blockage of the light penetration occurs, which decreases the degradation of MO^43^.

### Reusability

To check the reusability of the catalyst, Ag_0.04_ZrO_2_/rGO photocatalyst was washed with deionized water several times and dried in the oven after every experiment. Ag_0.04_ZrO_2_/rGO photocatalyst was used for the degradation of MO in five repeated experiments. In every experiment, the irradiation time was 100 min. The Ag_0.04_ZrO_2_/rGO photocatalyst exhibited a high visible light photostability after five repeated experiments, although a slight decrease of photocatalytic activity is observed compared to the first-run result from 87 to 78% degradation, respectively as shown in Fig. 8e,f.

### Phooelectrochemical measurements

**Figure 9:**
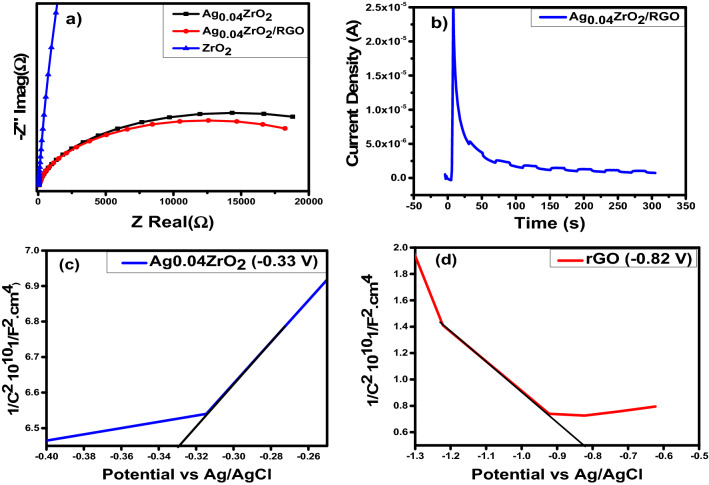
(**a**) Shows the EIS spectra of the prepared pure and heterostrucrure photocatalysts (**b**) shows chronoamperometric on/off study of the best Ag_0.04_ZrO_2_ /rGO photocatalyst. (**c**) Mott-Schottky plots of Ag_0.04_ZrO_2_ and (**d**) Mott-Schottky plots of rGO.

Figure 9a shows the electrochemical impedence spectroscopic measurements of the pure ZrO_2_, Ag_0.04_ZrO_2_ and Ag_0.04_ZrO_2_/rGO photocatalysts under visible light irradiation. The smallest semicircle is observed for the photocatalyst Ag_0.04_ZrO_2_/rGO, showing the lowest charge transfer resistance in the as prepared photocatalyst.

Chronoamperometric response is shown in Fig. 9b at a potential of 0.8 V under the chopped light illumination. The photocurrent increases immediately from OFF to ON state proving that the present system is sensitive to light illumination and efficient in the generation and separation of electron–hole pairs through p–n junction.

Figure 9c,d show the Mott-Schottky plots of Ag-doped ZrO_2_ and rGO. Flat band potential (Efb) is measured from these plots. The slope of Mott-Schottky plots of Ag_0.04_ZrO_2_ (− 0.32 V vs Ag/AgCl) is positive as compared to rGO (− 0.82 V vs Ag/AgCl) showing the n-type nature of Ag_0.04_ZrO_2_ and p-type nature of rGO, indicating the formation of an effective p–n junction between Ag_0.04_ZrO_2_ and rGO for the degradation of MO.

### Mechanisms of photocatalytic degradation of MO

The potential of the valence band and conduction band, as well as the band gap energy, are important factors to determine the mechanism. The potential of the conduction band was calculated from Mott-Schottky plots which is be − 0.12 eV vs RHE for Ag_0.04_ZrO_2_ and − 0.62 eV vs SHE for rGO. The bandgap energies calculated by using the Tauc plot are 3.11 eV for Ag_0.04_ZrO_2_ and 1.69 eV for rGO. The potential of the valence band of Ag_0.04_ZrO_2_ (2.99 eV) and rGO (1.07 eV) was calculated by using this formula: VB = CB + Eg.

**Figure 10:**
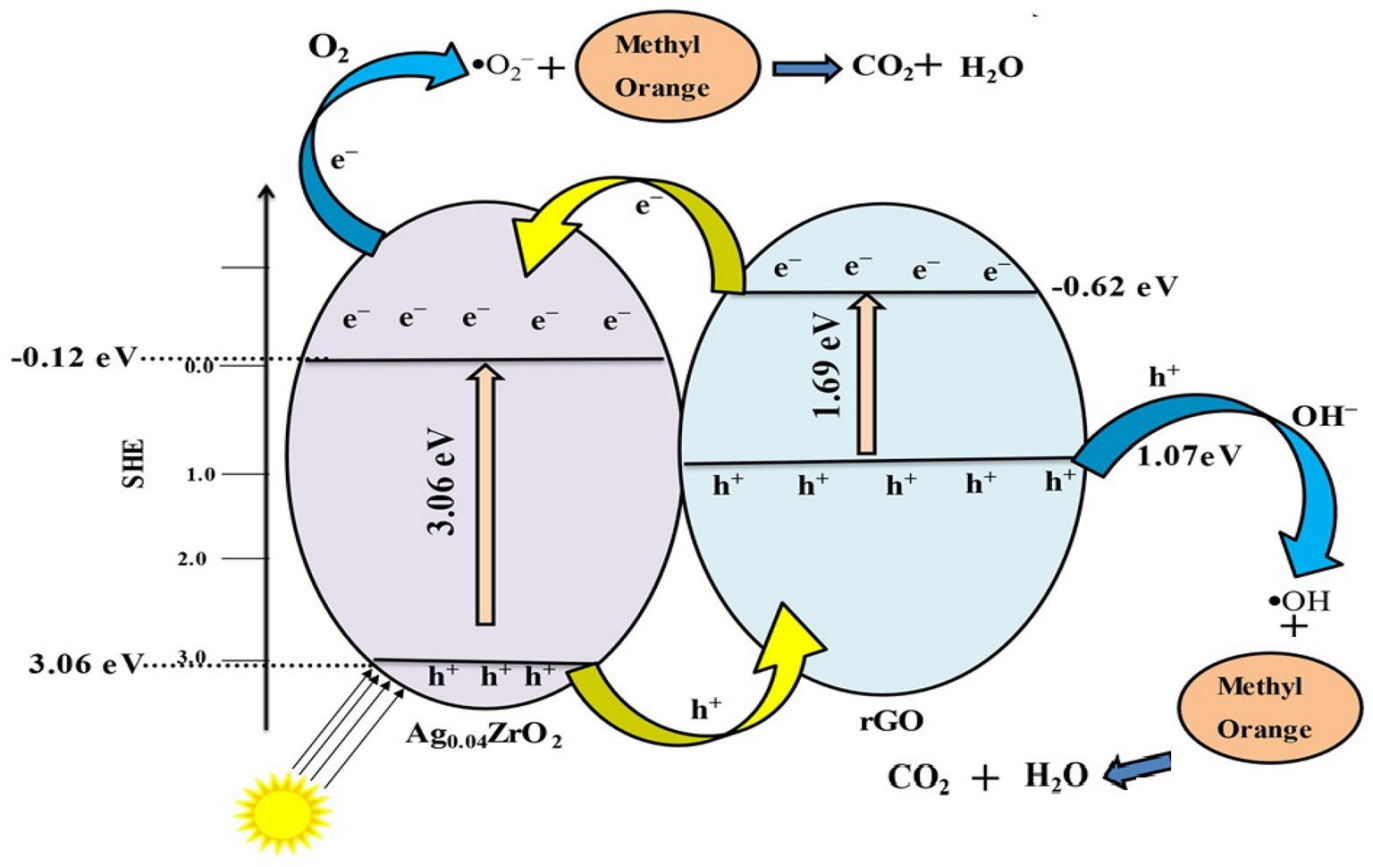
Mechanisms of photocatalytic degradation of MO by Ag_0.04_ZrO_2_/rGO.

The detailed mechanisms of photocatalytic degradation of MO by Ag_0.04_ZrO_2_/rGO are shown in Fig. 10. This mechanism shows that when light falls on the photocatalyst, the elctrons from the valence band of Ag_0.04_ZrO_2_ and rGO get excited and move to the conduction bands. The holes from the valence band of Ag_0.04_ZrO_2_ move the valence band of rGO. The electrons from the conduction band of rGO move to the conduction band of Ag_0.04_ZrO_2_ hence reducing the elecectron–hole recombination as shown by the PL spectra. These photoexcited electrons react with the adsorbed oxygen and convert it to superoxide radicals which react with methyl orange and immediately decompose the dye to water and CO_2_. A possible mechanistic rout is given below:

$$ {\text{Ag}}_{0.04} {\text{ZrO}}_{2} /{\text{rGO}} + {\text{light}} \to {\text{e}}^{ - } + {\text{h}}^{ + } $$

$$ {\text{e}}^{ - } + {\text{O}}_{2} \to \cdot {\text{O}}_{2}^{ - } $$

$$ \cdot {\text{O}}_{2}^{ - } + {\text{h}}^{ + } + {\text{MO}} \to {\text{CO}}_{2} + {\text{H}}_{2} {\text{O}} $$

Holes on the other hand react with the water molecules and produce OH^.^ radicals and react with methyl orange and immediadely decompose it to H_2_O and CO_2_. Therefore, this photocatalyst system provides active sites which shows the ability to harvest large amount of light hence better degradation efficiency. Many researchers have reported the degradation of methyl orange till now and a comparison table with the present study is shown in Table 2.

**Table 2:** A general comparison of the ZrO_2_ based photocatalyst for the degradation of MO from literature with the present study.

Sr.No	Photocatalyst	Efficiency/Time	Refs
1	Mn doped ZrO_2_	83%/100 min	^44^
2	Ag/TiO_2_-ZrO_2_	81.5%/90 min	^45^
3	TiO_2_/ZrO_2_	96%/180 min	^46^
4	TiO_2_-ZrO_2_	75.5%/150 min	^47^
5	Ag_0.04_ZrO_2_/rGO	87%/100 min	This study

## Conclusions

We have synthesized pure ZrO_2_, Ag-doped ZrO_2,_ and novel Ag-doped ZrO_2_/rGO photocatalysts by facile hydrothermal method. These photocatalysts were characterized by powder XRD, SEM, EDX, FTIR, photoluminescence (PL), UV–Vis diffuse reflectance (DRS), and Raman spectroscopy. The photodegradation of MO was studied with pure ZrO_2_, Ag-doped ZrO_2,_ and Ag-doped ZrO_2_/rGO photocatalysts at 100 min irradiation time under visible light. Reaction conditions were optimized for the best photocatalyst (Ag_0.04_ZrO_2_/rGO) by varying catalyst loading and pH of the solution. Ag_0.04_ZrO_2_/rGO exhibited the maximum photocatalytic degradation of MO (87%) as compared to Ag_0.04_ZrO_2_ (60%) and pure ZrO_2_ (26%) due to lower bandgap energy and a lower rate of recombination of e^−^/h^+^ pair. Reusability experiments showed the excellent stability of photocatalyst after five consecutive experiments. Hence, this is the first report on the facile hydrothermal synthesis of novel Ag_0.04_ZrO_2_/rGO photocatalyst for the degradation of methyl orange (MO).

## Methods

### Materials

All analytical grade chemicals were used as received without further purification. AgNO_3_ and Zr(NO_3_)_4_·5H_2_O were purchased from Sigma Aldrich. Deionized H_2_O was employed in all experiments.

### Synthesis of graphene oxide (GO)

GO was prepared by modified Hummers’ method^48^. Initially, H_2_SO_4_ (27 ml) was mixed with H_3_PO_4_ (3 ml) and stirred for several min. Then graphite powder (0.225 g) was added to the mixture and then added the KMnO_4_ (1.32 g) slowly. The mixture was stirred for 6 h until the color turns into dark green. Then H_2_O_2_ was added to the mixture and stirred for 10 min. After cooling HCl (10 ml) and H_2_O (30 ml) were added and centrifuge for 10 min. at 5000 rpm. The supernatant was removed and the residue was washed the HCl and H_2_O three times.

### Synthesis of zirconia (ZrO_2_)

ZrO_2_ was prepared by a simple hydrothermal method. Initially, a 0.01 M aqueous solution of zirconium nitrate was prepared and 25% ammonium hydroxide was added dropwise to the solution with constant stirring^49^. After 1 h, white precipitates were collected and transferred to Teflon lined autoclave. The precipitates were hydrothermally treated at 180 °C for 24 h. The product was obtained by centrifugation, washed several times with deionized water and ethanol, and dried at 80 °C in a vacuum oven.

### Synthesis of Ag_x_ZrO_2_

Ag-doped ZrO_2_ photocatalysts were prepared by hydrothermal method. Zirconium nitrate aqueous solution (0.01 M) was mixed with the 0.1 mM aqueous solution of silver nitrate. To the homogenized solution, 25% ammonium hydroxide was added dropwise with constant stirring. After 1 h, the white precipitates were collected and transferred to a Teflon-lined autoclave which was then hydrothermally treated at 180 °C for 24 h. The precipitates were separated by centrifuge, washed several times with deionized water and ethanol, and dried at 80 °C in a vacuum oven to obtain the product, Ag_0.01_ZrO_2_. The same experiment was repeated by increasing the concentration of silver nitrate (0.02 to 0.05 mM) to obtain Ag_0.02_ZrO_2_, Ag_0.03_ZrO_2_, Ag_0.04_ZrO_2_, and Ag_0.05_ZrO_2_.

### Synthesis of Ag_0.04_ZrO_2_/rGO photocatalyst

The photocatalyst, Ag_0.04_ZrO_2_/rGO (Ag_0.04_ZrO_2_: rGO in 1:1, 1:2, and 1:3 ratio) were prepared in situ by the procedure as discussed above. The aqueous solutions zirconium nitrate, silver nitrate was mixed with graphene oxide an autoclave and heated at 180 °C for 24 h. GO is thermally reduced to rGO under the reaction conditions^50^. The black precipitates of the nanocomposites were separated by centrifuge, washed several times with water and ethanol, and dried at 80 °C under vacuum.

### Characterization

X-ray diffractometer (DRONE-8, Russia), using Cu Kα radiation as the X-ray source, operated at 45 kV and 100 mA was utilized to study the crystalline structure and phase composition of photocatalysts. Scanning electron microscopy (MAIA3 TESCAN) was employed to determine the morphology of the photocatalysts. The absorbance of the photocatalysts was determined by utilizing the ultraviolet–visible (UV–Vis) diffuse reflectance spectroscopy (Lambda 365S, Perkin Elmer, Massachusetts, USA) in the wavelength range of 200–800 nm. Fourier transform infrared spectrometer (Alpha, Bruker) with range 550 to 4000 cm^−1^ was used to obtain IR spectra of the compound. Perkin Elmer spectrophotometer (Massachusetts, USA FL 6500/8500) with 150 W Xe lamp (200–900 nm) was used to measure the PL of photocatalysts.

### Degradation studies of methyl orange

The degradation studies of MO were performed with all prepared photocatalysts. The prepared photocatalysts (10 mg) were added in 100 mL of the aqueous solution of MO (10 ppm) and stirred initially for 30 min in the dark to attain adsorption–desorption equilibrium. Then the mixture was then exposed to UV–Visible light using a 500 W UV–Vis lamp. The 5 mL aliquot was taken every 20 min. and analyzed with UV–Vis spectrophotometer. The photocatalytic degradation treatment was studied at pH 3 and 100 min. irradiation time. The photocatalytic degradation efficiencies of photocatalysts were calculated using the following formula^51^:

$$ {\text{Degradation}}\;{\text{efficiency}}\;\left( \% \right) = \left( {{{(A_{0} - A)} \mathord{\left/ {\vphantom {{(A_{0} - A)} {A_{0} }}} \right. \kern-\nulldelimiterspace} {A_{0} }}} \right)100 $$

where *A*_*0*_ is the initial absorbance of MO solution and *A* is the absorbance after irradiation.

## Supplementary Information

Supplementary Information.

## Data Availability

All data generated or analyzed during this study are included in this article and its supplementary information file.

## References

[1] Vattikuti SVP, Byon C, Reddy CV (2016). ZrO 2/MoS 2 heterojunction photocatalysts for efficient photocatalytic degradation of methyl orange. Electron. Mater. Lett..

[2] Chan SH, Yeong WuT, Juan JC, Teh CY (2011). Recent developments of metal oxide semiconductors as photocatalysts in advanced oxidation processes (AOPs) for treatment of dye waste-water. J. Chem. Technol. Biotechnol..

[3] Katsuda T, Ooshima H, Azuma M, Kato J (2006). New detection method for hydrogen gas for screening hydrogen-producing microorganisms using water-soluble Wilkinson's catalyst derivative. J. Biosci. Bioeng..

[4] Biswas P, Wu C-Y (2005). Nanoparticles and the environment. J. Air Waste Manag. Assoc..

[5] Li S, Han Q, Jia X, Zahid AH, Bi H (2020). Room-temperature one-step synthesis of tube-like S-scheme BiOBr/BiO (HCOO) Br-x heterojunction with excellent visible-light photocatalytic performance. Appl. Surf. Sci..

[6] Emam HE, Ahmed HB, Gomaa E, Helal MH, Abdelhameed RM (2020). Recyclable photocatalyst composites based on Ag3VO4 and Ag2WO4@ MOF@ cotton for effective discoloration of dye in visible light. Cellulose.

[7] Shahabuddin S, Sarih NM, Ismail FH, Shahid MM, Huang NM (2015). Synthesis of chitosan grafted-polyaniline/Co 3 O 4 nanocube nanocomposites and their photocatalytic activity toward methylene blue dye degradation. RSC Adv..

[8] Wang Y (2020). Synthesizing Co3O4-BiVO4/g-C3N4 heterojunction composites for superior photocatalytic redox activity. Sep. Purif. Technol..

[9] Zhang H (2019). Construction of a novel BON-Br-AgBr heterojunction photocatalysts as a direct Z-scheme system for efficient visible photocatalytic activity. Appl. Surf. Sci..

[10] Shi W (2021). Carbon dots anchored high-crystalline gC 3 N 4 as a metal-free composite photocatalyst for boosted photocatalytic degradation of tetracycline under visible light. J. Mater. Sci..

[11] Shi W (2020). Dual enhancement of capturing photogenerated electrons by loading CoP nanoparticles on N-deficient graphitic carbon nitride for efficient photocatalytic degradation of tetracycline under visible light. Sep. Purif. Technol..

[12] Guo F (2021). Construction of Cu3P-ZnSnO3-g-C3N4 pnn heterojunction with multiple built-in electric fields for effectively boosting visible-light photocatalytic degradation of broad-spectrum antibiotics. Sep. Purif. Technol..

[13] Shi W (2020). Fabrication of ternary Ag3PO4/Co3 (PO4) 2/g-C3N4 heterostructure with following Type II and Z-Scheme dual pathways for enhanced visible-light photocatalytic activity. J. Hazard. Mater..

[14] Chen F, Zou W, Qu W, Zhang J (2009). Photocatalytic performance of a visible light TiO2 photocatalyst prepared by a surface chemical modification process. Catal. Commun..

[15] Wang J, Fan H, Yu H (2016). Synthesis of hierarchical flower-like SnO2 nanostructures and their photocatalytic properties. Optik.

[16] Liu J (2015). 3D flowerlike α-Fe2O3@ TiO2 core–shell nanostructures: General synthesis and enhanced photocatalytic performance. ACS Sustain. Chem. Eng..

[17] Abdelkader E, Nadjia L, Naceur B, Noureddine B (2016). SnO2 foam grain-shaped nanoparticles: Synthesis, characterization and UVA light induced photocatalysis. J. Alloy. Compd..

[18] Singh R, Pal B (2015). Woolen bun shaped CdS microspheres enfolded 1D nanowires for the superior photooxidation of dyes: A comparative case study. J. Mol. Catal. A: Chem..

[19] Vattikuti SP, Byon C, Reddy CV (2015). Synthesis of MoS2 multi-wall nanotubes using wet chemical method with H2O2 as growth promoter. Superlattices Microstruct..

[20] Wang Q, Li J, Zhang W, Zhong M (2020). Plasma-assisted synthesis of bicrystalline ZnS nanobelts with enhanced photocatalytic ability. Electron. Mater. Lett..

[21] Vattikuti SP, Byon C, Chitturi V (2016). Selective hydrothermally synthesis of hexagonal WS2 platelets and their photocatalytic performance under visible light irradiation. Superlattices Microstruct..

[22] Yang R, Zhong S, Zhang L, Liu B (2020). PW12/CN@ Bi2WO6 composite photocatalyst prepared based on organic-inorganic hybrid system for removing pollutants in water. Sep. Purif. Technol..

[23] Singhania A, Gupta SM (2017). Nanocrystalline ZrO2 and Pt-doped ZrO2 catalysts for low-temperature CO oxidation. Beilstein J. Nanotechnol..

[24] Yang R (2020). One-step preparation (3D/2D/2D) BiVO4/FeVO4@rGO heterojunction composite photocatalyst for the removal of tetracycline and hexavalent chromium ions in water. Chem. Eng. J..

[25] Imtiaz F, Rashid J, Xu M (2019). Concepts of Semiconductor Photocatalysis.

[26] Yang R, Zhao Q, Liu B (2020). Two-step method to prepare the direct Z-scheme heterojunction hierarchical flower-like Ag@ AgBr/Bi2MoO6 microsphere photocatalysts for waste water treatment under visible light. J. Mater. Sci.: Mater. Electron..

[27] Liu B (2018). Construction of fiber-based BiVO4/SiO2/reduced graphene oxide (RGO) with efficient visible light photocatalytic activity. Cellulose.

[28] Corami A, Mignardi S, Ferrini V (2007). Copper and zinc decontamination from single-and binary-metal solutions using hydroxyapatite. J. Hazard. Mater..

[29] Isacfranklin M (2020). Synthesis of highly active biocompatible ZrO2 nanorods using a bioextract. Ceram. Int..

[30] Li L, Wang W (2003). Synthesis and characterization of monoclinic ZrO2 nanorods by a novel and simple precursor thermal decomposition approach. Solid State Commun..

[31] Wang Z (2013). Hydrothermal synthesis and humidity sensing properties of size-controlled zirconium oxide (ZrO2) nanorods. J. Colloid Interface Sci..

[32] Tyagi B, Sidhpuria K, Shaik B, Jasra RV (2006). Synthesis of nanocrystalline zirconia using sol−gel and precipitation techniques. Ind. Eng. Chem. Res..

[33] Dhorabe PT, Lataye DH, Ingole RS (2017). Adsorptive removal of 4-nitrophenol from aqueous solution by activated carbon prepared from waste orange peels. J. Hazard. Toxic Radioact. Waste.

[34] Lu Z (2018). Facile microwave synthesis of a Z-scheme imprinted ZnFe2O4/Ag/PEDOT with the specific recognition ability towards improving photocatalytic activity and selectivity for tetracycline. Chem. Eng. J..

[35] López R, Gómez R (2012). Band-gap energy estimation from diffuse reflectance measurements on sol–gel and commercial TiO 2: A comparative study. J. Sol-Gel. Sci. Technol..

[36] Yu X (2018). Enhanced photocatalytic activity of Ag–ZnO/RGO nanocomposites for removal of methylene blue. J. Mater. Sci.: Mater. Electron..

[37] Salavati H, Tavakkoli N, Hosseinpoor M (2012). Preparation and characterization of polyphosphotungstate/ZrO2 nanocomposite and their sonocatalytic and photocatalytic activity under UV light illumination. Ultrason. Sonochem..

[38] Farhadi S, Zaidi M (2009). Polyoxometalate–zirconia (POM/ZrO2) nanocomposite prepared by sol–gel process: A green and recyclable photocatalyst for efficient and selective aerobic oxidation of alcohols into aldehydes and ketones. Appl. Catal. A.

[39] Lai L-J (2005). Photoluminescence of zirconia films with VUV excitation. J. Electron Spectrosc. Relat. Phenom..

[40] Singh G (2012). ZnO decorated luminescent graphene as a potential gas sensor at room temperature. Carbon.

[41] Ding J (2014). Photoluminescence investigation about zinc oxide with graphene oxide & reduced graphene oxide buffer layers. J. Colloid Interface Sci..

[42] Li J (2008). The removal of MO molecules from aqueous solution by the combination of ultrasound/adsorption/photocatalysis. Ultrason. Sonochem..

[43] Chen M, Chu W (2012). Efficient degradation of an antibiotic norfloxacin in aqueous solution via a simulated solar-light-mediated Bi2WO6 process. Ind. Eng. Chem. Res..

[44] Reddy CV (2019). Mn-doped ZrO2 nanoparticles prepared by a template-free method for electrochemical energy storage and abatement of dye degradation. Ceram. Int..

[45] Li L, Lu D, Ji Y, Zhao Y-H (2010). Preparation of nanocomposite Ag/TiO2-ZrO2 and the microwave enhanced photocatalytic degradation of methyl orange. Acta Physico-Chim. Sin..

[46] Qu X, Xie D, Cao L, Du F (2014). Synthesis and characterization of TiO2/ZrO2 coaxial core–shell composite nanotubes for photocatalytic applications. Ceram. Int..

[47] Ruíz-Santoyo V, Marañon-Ruiz VF, Romero-Toledo R, González Vargas OA, Pérez-Larios A (2021). Photocatalytic degradation of rhodamine b and methylene orange using TiO2-ZrO2 as nanocomposite. Catalysts.

[48] Zaaba NI (2017). Synthesis of graphene oxide using modified hummers method: Solvent influence. Procedia Eng..

[49] Sampurnam S (2019). Synthesis and characterization of Keggin-type polyoxometalate/zirconia nanocomposites: Comparison of its photocatalytic activity towards various organic pollutants. J. Photochem. Photobiol. A.

[50] Vallés C, David Núñez J, Benito AM, Maser WK (2012). Flexible conductive graphene paper obtained by direct and gentle annealing of graphene oxide paper. Carbon.

[51] Sakthivel B, Mohan R, Ravichandran K, Nithya A, Jothivenkatachalam K, Ravidhas C (2014). Influence of spray flux density on thephotocatalytic activity and certain physicalproperties of ZnO thin films. J. Mater. Sci.: Mater. Electron..

