# Supplementary material for: Development of Ag0.04ZrO2/rGO heterojunction, as an efficient visible light photocatalyst for degradation of methyl orange
Source: Sci Rep. 2022 Jul 19;12:12308. doi: 10.1038/s41598-022-16673-7 (PMC9296493; doi:10.1038/s41598-022-16673-7)
Supplement: Supplementary file 1 — Supplementary Information. [file 41598_2022_16673_MOESM1_ESM.docx]

**Development of Ag_0.04_ZrO_2_/rGO Heterojunction, as an Efficient Visible Light Photocatalyst for Degradation of Methyl Orange**

Rana Muhammad Arslan Iqbal^1^, Tehmina Akhtar^1^, Effat Sitara^1^, Habib Nasir^*󠄀1^, Aliya Fazal^2^, Uzaira Rafique^3^, Sharif Ullah^1^, and Adeel Mehmood^4^

^1^ Department of Chemistry, School of Natural Sciences, National University of Sciences and Technology, H-12, Islamabad 44000, Pakistan

^2^ Department of Chemistry, Fatima Jinnah Women University, Rawalpindi, Pakistan

^3^ Department of Environmental Sciences, Fatima Jinnah Women University, Rawalpindi, Pakistan

^4^ Department of Chemical Engineering and Energy Systems Research, Ajou University, Suwon-si, Gyeonggi-do 16499, Republic of Korea

Email corresponding author: *󠄀habibnasir@sns.nust.edu.pk


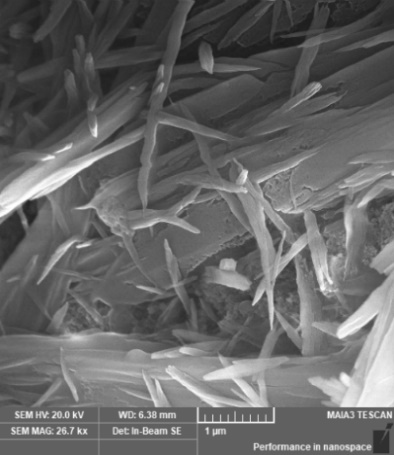

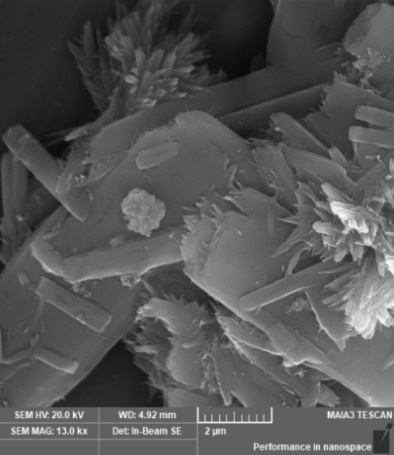

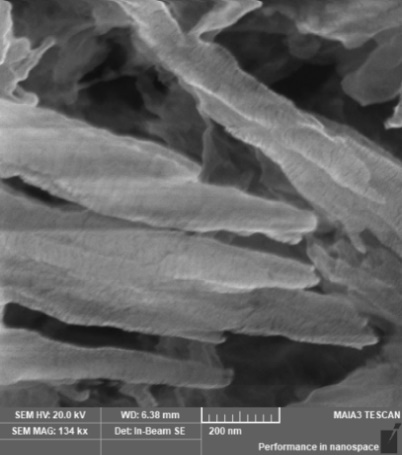


**a**

**b**

**c**


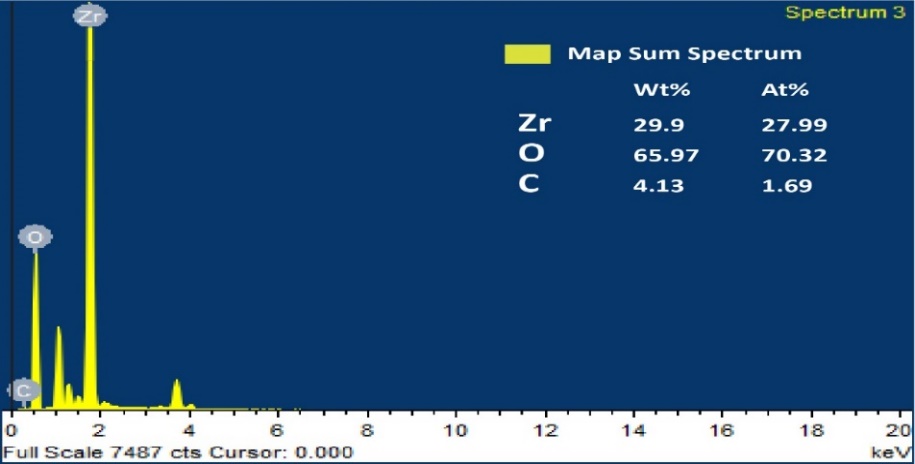


**d**

**Fig. S1 Scanning electron micrograph of ZrO_2_ (a-c) EDX of ZrO_2_(d).**


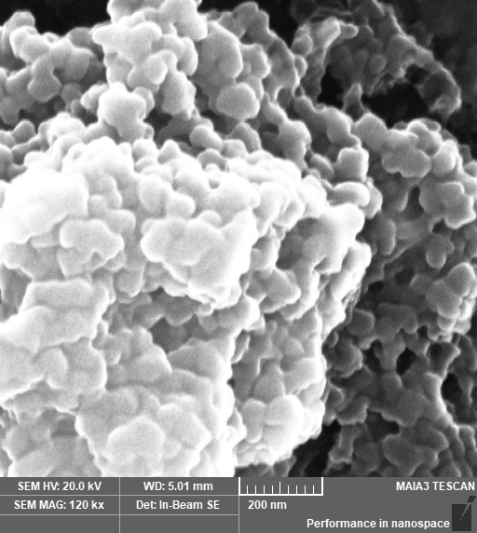

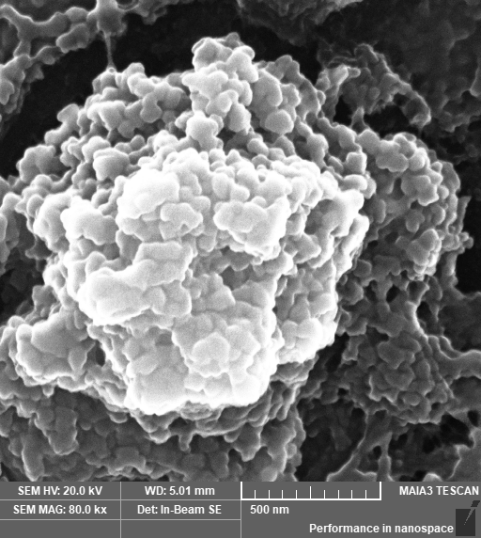

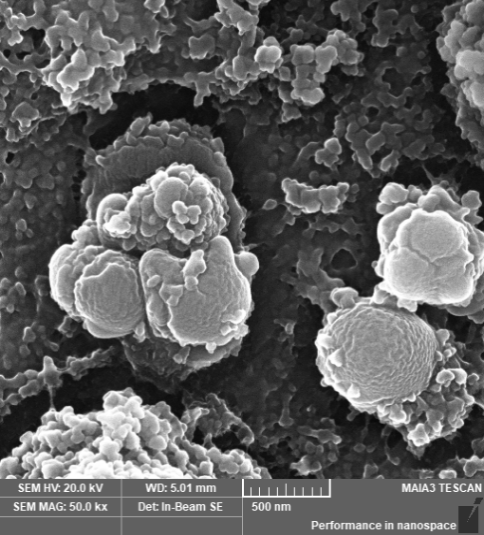

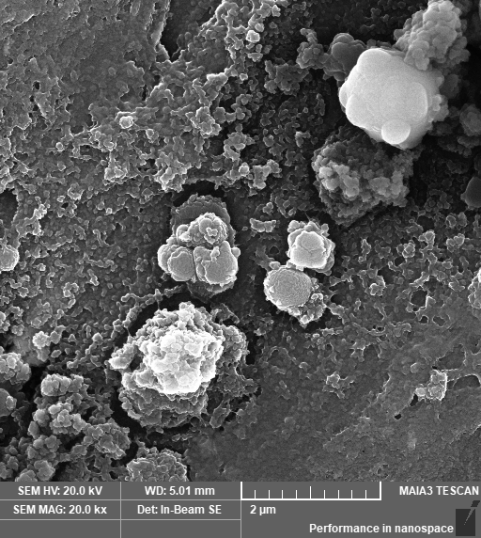


**a**

**b**

**c**

**d**


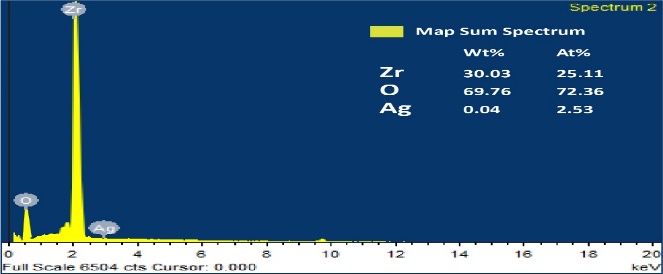


**e**

**Fig. S2 Scanning electron micrograph of Ag_0.04_ZrO_2_ (a-d) EDX of Ag_0.04_ZrO_2_(e).**

**Fig. S3 FTIR spectra of, a) ZrO_2_ and Ag_0.04_ ZrO_2_ and b) Ag_0.04_ZrO_2_/rGO (1:1).**


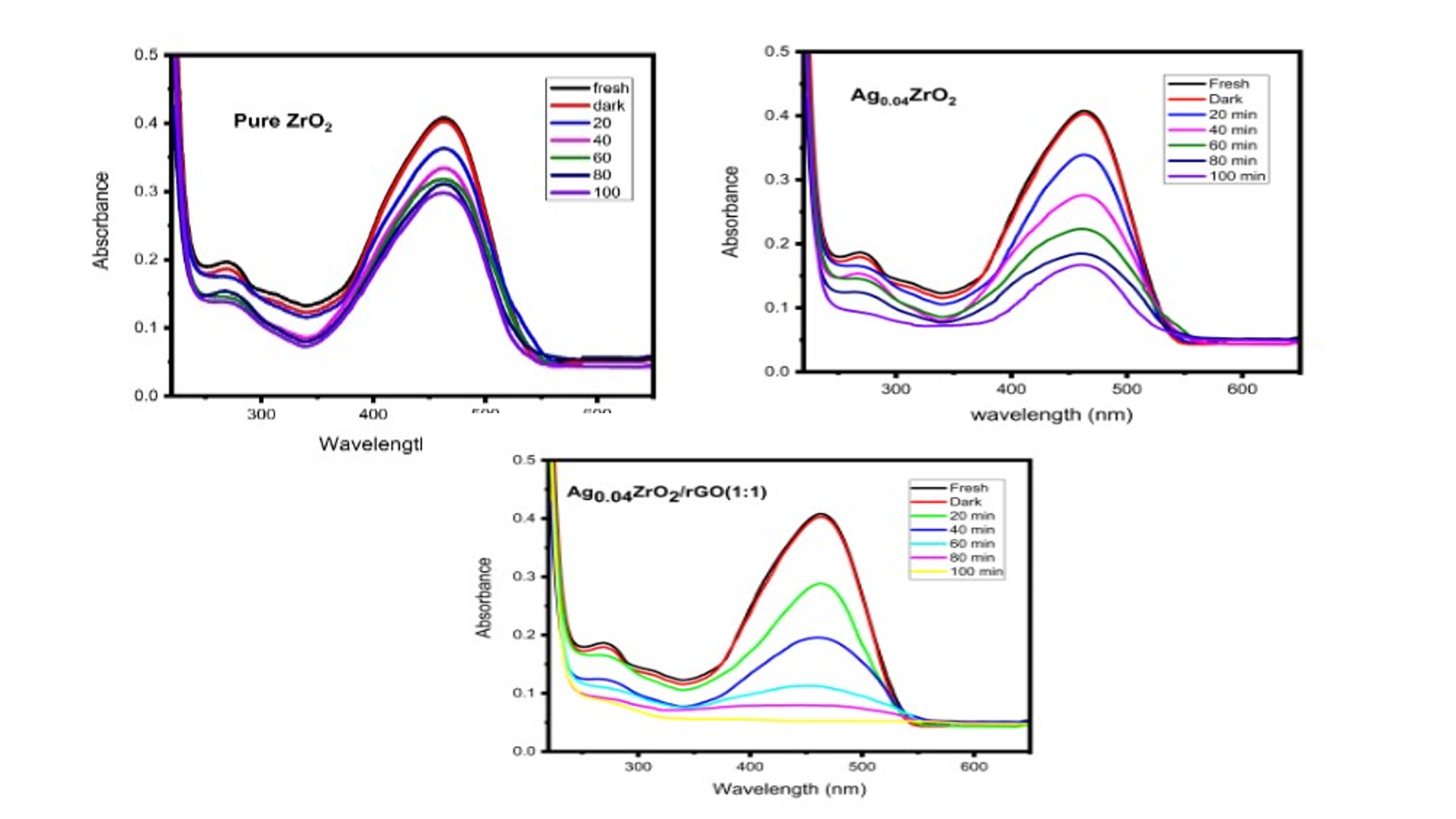


**c**

**b**

**a**

**Fig. S4 Comparison of degradation of MO (a)ZrO_2_ (b) Ag_0.04_ZrO_2_ (c) Ag_0.04_ZrO_2_/rGO.**





**Fig. S5 Comparison of degradation verses time of MO of ZrO_2_, Ag_0.04_ZrO_2_ and Ag_0.04_ZrO_2_/rGO.**


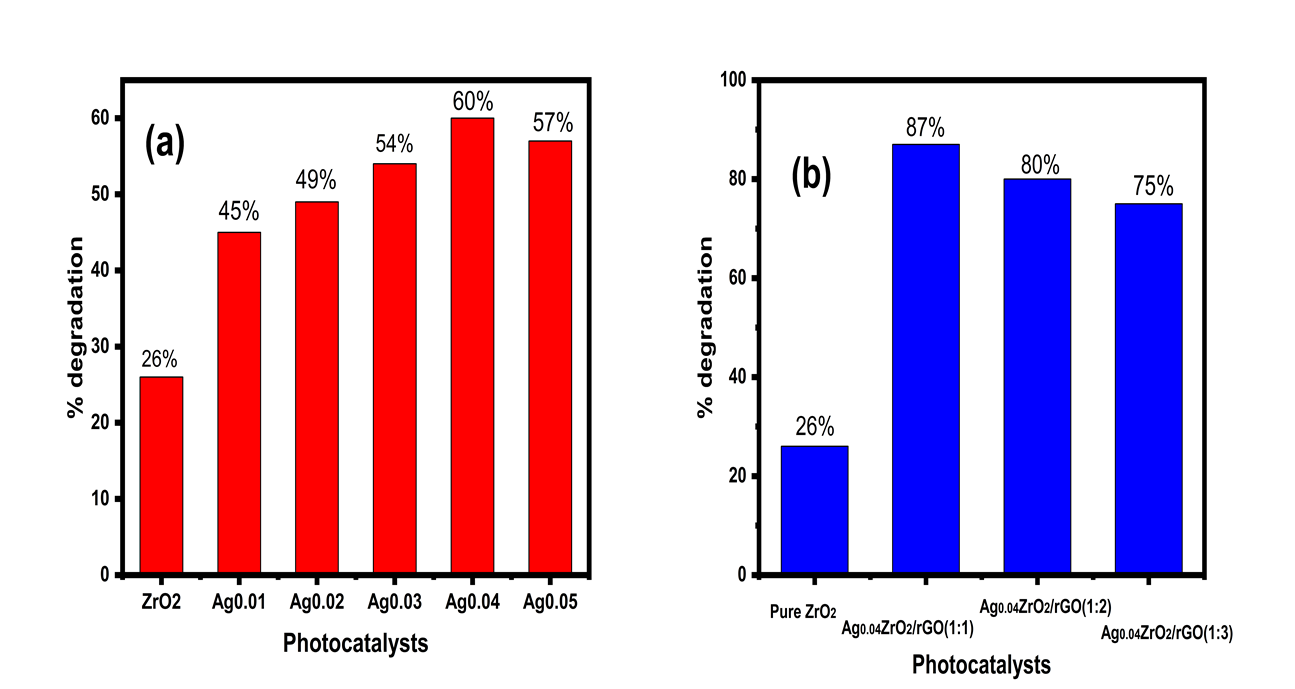


**Fig. S6 Comparison of % degradation of MO (a)ZrO_2_ and Ag_x_ZrO_2_ x=0.01 to 0.05 (b) ZrO_2_ and Ag_0.04_ZrO_2_/ rGO (1:1, 1:2 and 1:3) photocatalysts.**

**
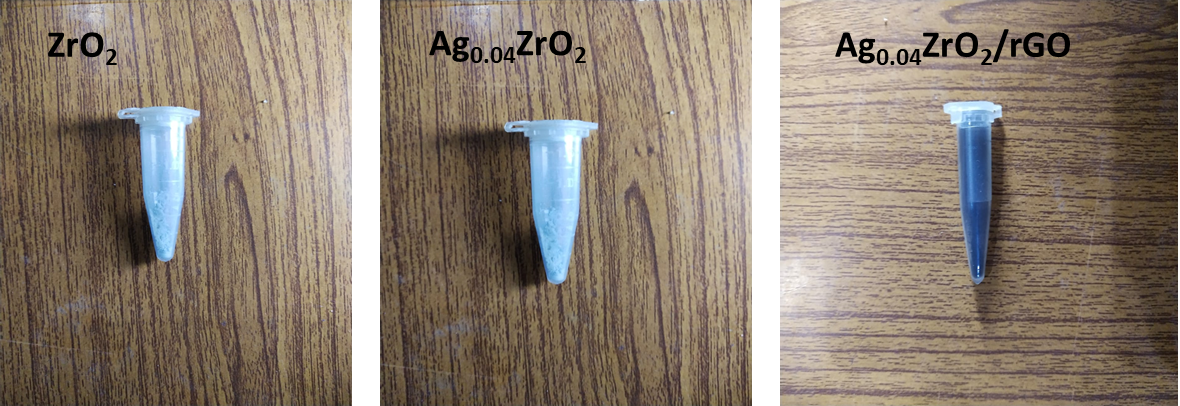
**

**Fig. S7 Digital photo of actual samples.**

**Fig. S8 EPR spectrum of composite Ag_0.04_ZrO_2_/rGO.**

**Fig. S9 XRD of the best photocatalyst Ag_0.04_ZrO_2_/rGO before and after degradation experiment.**


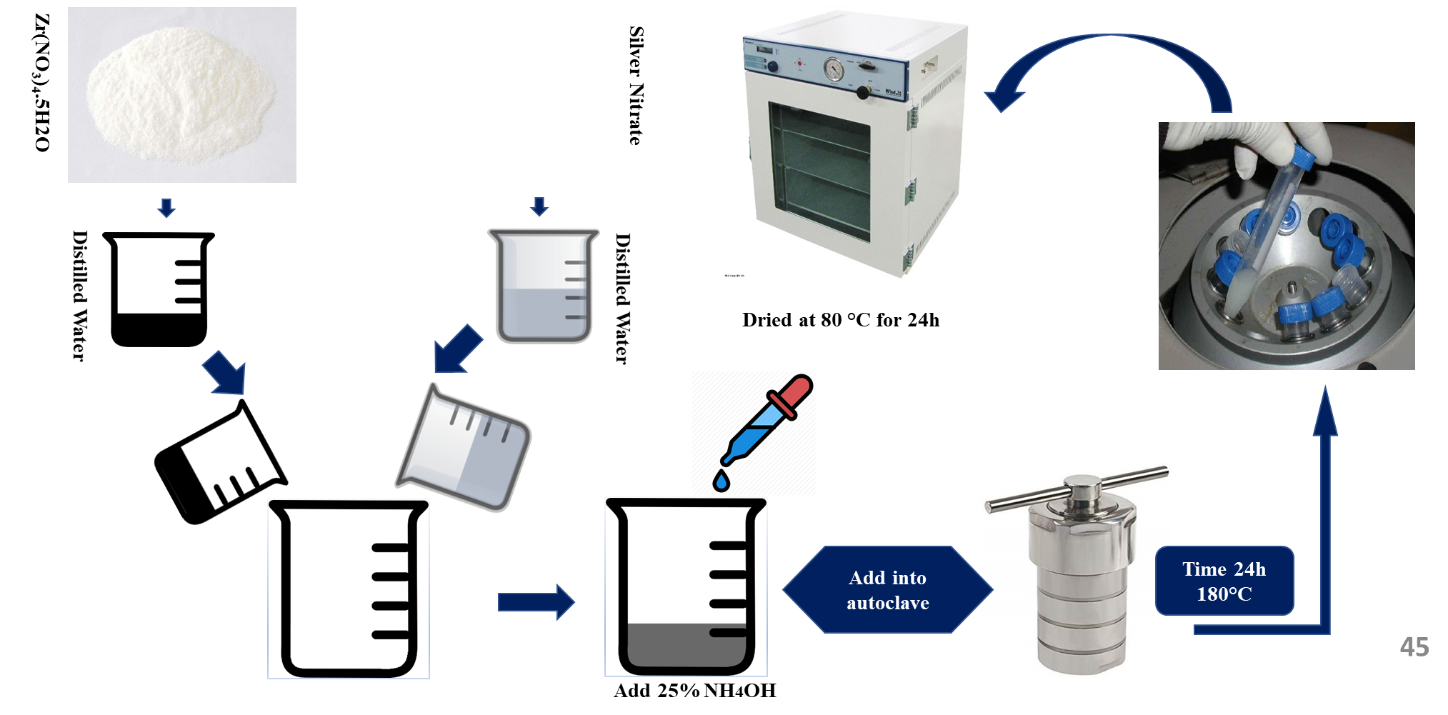


**Fig. S10 Scheme of the experimental setup.**

**Fig. S11 Apparent rate constants of ZrO_2_, Ag@ZrO_2_ and composites Ag@ZrO_2_/rGO.**
